# Plasma IgG to Linear Epitopes in the V2 and V3 Regions of HIV-1 gp120 Correlate with a Reduced Risk of Infection in the RV144 Vaccine Efficacy Trial

**DOI:** 10.1371/journal.pone.0075665

**Published:** 2013-09-26

**Authors:** Raphael Gottardo, Robert T. Bailer, Bette T. Korber, S. Gnanakaran, Joshua Phillips, Xiaoying Shen, Georgia D. Tomaras, Ellen Turk, Gregory Imholte, Larry Eckler, Holger Wenschuh, Johannes Zerweck, Kelli Greene, Hongmei Gao, Phillip W. Berman, Donald Francis, Faruk Sinangil, Carter Lee, Sorachai Nitayaphan, Supachai Rerks-Ngarm, Jaranit Kaewkungwal, Punnee Pitisuttithum, James Tartaglia, Merlin L. Robb, Nelson L. Michael, Jerome H. Kim, Susan Zolla-Pazner, Barton F. Haynes, John R. Mascola, Steve Self, Peter Gilbert, David C. Montefiori

**Affiliations:** 1 Fred Hutchinson Cancer Research Center, Seattle, Washington, United States of America; 2 Vaccine Research Center, National Institutes of Allergy and Infectious Diseases, National Institutes of Health, Bethesda, Maryland, United States of America; 3 Theoretical Biology and Biophysics, Los Alamos National Laboratory, Los Álamos, New Mexico, United States of America; 4 Duke University Medical Center, Durham, North Carolina, United States of America; 5 JPT Peptide Technologies GmbH, Berlin, Germany; 6 Baskin School of Engineering, University of California Santa Cruz, Santa Cruz, California, United States of America; 7 Global Solutions for Infectious Diseases, South San Francisco, California, United States of America; 8 Department of Retrovirology, US Army Medical Component, AFRIMS, Bangkok, Thailand; 9 Department of Disease Control, Ministry of Public Health, Nonthaburi, Thailand; 10 Center of Excellence for Biomedical and Public Health Informatics BIOPHICS, Faculty of Tropical Medicine, Mahidol University, Bangkok, Thailand; 11 Vaccine Trial Center and Department of Clinical Tropical Medicine, Mahidol University, Bangkok, Thailand; 12 Department of Research and Development, Sanofi Pasteur, Swiftwater, Pennsylvania, United States of America; 13 US Military HIV Research Program, Walter Reed Army Institute of Research, Silver Spring, Maryland, United States of America; 14 Veterans Affairs New York Harbor Healthcare System, New York, New York, United States of America; 15 New York University School of Medicine, New York, New York, United States of America; The University of Hong Kong, Hong Kong

## Abstract

Neutralizing and non-neutralizing antibodies to linear epitopes on HIV-1 envelope glycoproteins have potential to mediate antiviral effector functions that could be beneficial to vaccine-induced protection. Here, plasma IgG responses were assessed in three HIV-1 gp120 vaccine efficacy trials (RV144, Vax003, Vax004) and in HIV-1-infected individuals by using arrays of overlapping peptides spanning the entire consensus gp160 of all major genetic subtypes and circulating recombinant forms (CRFs) of the virus. In RV144, where 31.2% efficacy against HIV-1 infection was seen, dominant responses targeted the C1, V2, V3 and C5 regions of gp120. An analysis of RV144 case-control samples showed that IgG to V2 CRF01_AE significantly inversely correlated with infection risk (OR= 0.54, p=0.0042), as did the response to other V2 subtypes (OR=0.60-0.63, p=0.016-0.025). The response to V3 CRF01_AE also inversely correlated with infection risk but only in vaccine recipients who had lower levels of other antibodies, especially Env-specific plasma IgA (OR=0.49, p=0.007) and neutralizing antibodies (OR=0.5, p=0.008). Responses to C1 and C5 showed no significant correlation with infection risk. In Vax003 and Vax004, where no significant protection was seen, serum IgG responses targeted the same epitopes as in RV144 with the exception of an additional C1 reactivity in Vax003 and infrequent V2 reactivity in Vax004. In HIV-1 infected subjects, dominant responses targeted the V3 and C5 regions of gp120, as well as the immunodominant domain, heptad repeat 1 (HR-1) and membrane proximal external region (MPER) of gp41. These results highlight the presence of several dominant linear B cell epitopes on the HIV-1 envelope glycoproteins. They also generate the hypothesis that IgG to linear epitopes in the V2 and V3 regions of gp120 are part of a complex interplay of immune responses that contributed to protection in RV144.

## Introduction

The efficacy of most licensed vaccines is associated with pathogen-specific antibody (Ab) responses as measured by either virus neutralization or antigen binding [1]. Most interest for HIV-1 vaccines has focused on virus neutralization [2], an emphasis that is based in part on the ability of passively transferred neutralizing Abs to prevent infection after experimental AIDS virus challenge in non-human primates [3-5]. A number of broadly neutralizing Abs (bnAbs) have been identified that would be desirable to induce with HIV-1 vaccines [6]. Some bnAbs target discontinuous conformational epitopes on the surface gp120 [7-18], while others target a set of linear epitopes in the membrane-proximal external region (MPER) of the transmembrane gp41 [19-21]. Additional epitopes are present on defective envelope (Env) glycoprotein spikes of the virus [22] and on the surface of infected cells [23] that can serve as targets for non-neutralizing Abs whose Fc receptor (FcR)-mediated antiviral effector functions might be beneficial for vaccines [24–29]. Little is known about the epitopes of non-neutralizing Abs that possess these functions.

Non-neutralizing Abs are gaining attention for HIV-1 vaccines because of the modest 31.2% protection against the acquisition of HIV-1 infection in the RV144 Thai trial [30]. Virus-specific CD8^+^ T cell responses were very weak in this trial [30], as was the neutralizing Ab response, which did not appear to target Tier 2 circulating strains of the virus [31]. A correlates study found a lower risk of HIV-1 infection in RV144 vaccine recipients whose plasma IgG bound an antigen comprising the gp120 variable regions 1 and 2 (V1V2) attached to the C-terminus of a murine leukemia virus (MLV) gp70 scaffold (gp70-V1V2) [32]. Subsequent studies with cyclic and linear peptides showed that V2-specific serum Abs in RV144 target the mid-loop region of V2 comprising gp120 amino acids 165-184, with a major dependency on lysine (K) at position 169 and valine (V) at position 172 [33,34]. Complementing these observations, a genetic sieve analysis of breakthrough viruses in RV144 found increased vaccine efficacy against viruses containing K169, which is also present in the CRF01_AE vaccine strains [35]. Two monoclonal Abs (CH 58 and CH 59) from RV144 vaccine recipients recognize this same region on linear V2 peptides, have a strict requirement for K169, bind HIV-1-infected cells and mediate antibody-dependent cellular cytotoxicity (ADCC) activity, but do not neutralize Tier 2 strains of HIV-1 [36].

Given the potential importance of non-neutralizing antibodies that bind linear peptides, we performed a systematic analysis of Env peptide binding Abs in RV144 and in two HIV-1 vaccine efficacy trials (Vax003, Vax004) where no significant protection was seen [37,38]. For comparison, we also examined the response in chronically HIV-1 infected subjects. Env-specific IgG was assessed with arrays of overlapping peptides spanning the entire consensus gp160 of all major genetic subtypes and circulating recombinant forms (CRFs) of HIV-1.

## Materials and Methods

### Ethics Statement

This study utilized pre-existing, de-identified specimens and was conducted under the approval of the local Institutional Review Boards (IRBs). The following IRBs conducted oversight for their respective sites: RV144- Ministry of Public Health (Bangkok, Thailand), Royal Thai Army (Thailand), Mahidol University (Bangkok, Thailand); Vax003 - The Bangkok Metropolitan Administration (Tropical Medicine of Mahidol University & HIV/AIDS Collaboration (Bangkok, Thailand); Vax004- Colorado Multiple Institution Review Board (Denver, CO), Saint Louis University (St Louis, MO), Johns Hopkins School of Medicine (Baltimore, MD), Fenway Community Health Center (Boston ,MA), Philadelphia Fight (Philadelphia, PA), Chicago Center for Clinical Research (Chicago, IL), AIDS Research Alliance (West Hollywood, CA), Louisiana State University Medical Center (New Orleans, LA), University of Rochester (Rochester, NY), Infectious Disease Research Institute, Inc. (Tampa, FL), Clinical Research Puerto Rico (San Jaun, PR), University of California, Irvine (Irvine, CA), University of California, San Francisco (San Francisco, CA), University of Washington (Seattle, WA), Hennepin County Medical Center (Minneapolis, MN), The Mount Sinai Medical Center (New York, NY), University Medical Center of southern Nevada (Las Vegas, NV), University of New Mexico (Albuquerque, NM), University of Illinois at Chicago Medical Center (Chicago, IL), Abbott Northwestern Hospital (Minneapolis, MN), St. John’s Doctors Building (Tulsa, OK), Dutchess County Dept of Health (Poughkeepsie, NY), New York Blood Center (New York, NY), New York Medical Center and Bellevue Hospital Center (New York, NY), Howard Brown Health Center (Chicago, IL), The Ohio State University (Columbus, OH), The University of Texas Medical Branch at Galveston (Galveston, TX), Kansas City AIDS Research Consortium (Kansas City, MO), University of California, Davis (Davis, CA), Central Florida Research Initiative (Maitland, FL), Community AIDS Resource, Inc (Coral Gables, FL), Palm Beach Research Center (West Palm Beach, FL), University of Hawaii (Honolulu, HI), AIDS Research Consortium of Atlanta, Inc (Atlanta, GA), University of California, San Francisco (San Francisco, CA), Erie County Medical Center (Buffalo, NY), ViRx Inc. (Palm Spring, CA), Municipal Health Services, Dept of Public Health and Environment (Amsterdam, Netherlands), Santa Public Health Dept. (San Jose, CA), Arizona Clinical Research Center, Inc. (Tucson, AZ), Albany Medical College (Albany, NY), New Jersey Community Research Initiative (Newark, NJ), Duval County Health Department (Jacksonville, FL), University Hospitals of Cleveland (Cleveland, OH), Oak Lawn Physicians Group (Dallas, TX), Nelson-Tebedo Community Clinic (Dallas, TX), The University of Alabama at Birmingham (Birmingham, AL), Community Hospitals Indianapolis (Indianapolis, IN), PW Clinical Research, LLC (Winston-Salem, NC), St. Paul’s Hospital (Vancouver British Columbia, Canada), Hospital Saint-Luc du CHUM (Montréal, Canada), Omaga Medical Research (Providance, RI), Wisconsin AIDS Research Consortium (Milwaukee, WI), The Research & Education Group (Portland, OR), University of Pittsburgh (Pittsburgh, PA), Phoenix Body Positive, Inc. (Phoenix, AZ), The Miriam Hospital (Providance, RI), Nalle Clinic (Charlotte, NC), Memorial Hospital of Rhode Island (Pawtucket, RI), Canadian HIV Trials Network (Toronto, ON, Canada); HIV-1-infected subjects- Duke University Medical Center (Durham, NC), Siriraj Ethics Committee (Bangkok, Thailand), Beth Israel Deaconess Medical Center (Boston, Massachusetts), Queen Mary’s School of Medicine and Dentistry (United Kingdom), Division of Human Subject Protection, Walter Reed Army Institute of Research (DHSP-WRAIR) (Bangkok, Thailand), Institutional Review Board for Chinese Center for Disease Control and Prevention/National Center for AIDS/STD Control and Prevention (Beijing, China), University of Witwatersrand – Human Research Ethics Committee (Human) (Johannesburg, South Africa), HIV/AIDS Research Committee of The Uganda National Council for Science and Technology (Kampala, Uganda). The data were analyzed anonymously.

### Specimens

Serum and plasma samples were obtained from the RV144, Vax003 and Vax004 HIV-1 vaccine efficacy trials (registration numbers NCT00223080, NCT0006327 and NCT00002441, respectively, ClinicalTrials.gov). RV144 tested two inoculations (weeks 0, 4) with a recombinant canarypox vector (vCP1521) expressing Gag and Pro of HIV-1 MN (subtype B), and membrane-linked gp120 from strain 92TH023 (CRF01_AE), followed by two boosts at weeks 12 and 24 with vCP1521 plus bivalent gp120 protein (AIDSVAX B/E, MN and A244 strains) in a community-based heterosexual population in Thailand [17]. Plasma samples were obtained pre-immunization (week 0) and 2 weeks after the final inoculation (week 26) from 41 vaccine recipients (cases) who acquired HIV-1 infection after week 26, and from an additional 205 vaccine recipients (controls) selected randomly among those who had not acquire infection by the end of the trial (month 42) [32]. Vax003 tested seven inoculations with bivalent gp120 protein (AIDSVAX B/E, weeks 0, 1, 6, 12, 18, 24, and 36) in a cohort of mostly injection drug using men in Thailand [37]. Vax004 tested seven inoculations with bivalent gp120 protein (AIDSVAX B/B, MN and GNE8 strains) at weeks 0, 1, 6, 12, 18, 24, and 30 in mostly men who have sex with men in North America and Europe [38]. Peak antibody responses in both trials were observed 2 weeks after the fourth inoculation (month 12.5) [37,39]. Serum samples from Vax003 and Vax004 were obtained at baseline and month 12.5 from 90 vaccine recipients in Vax003 and from 20 vaccine recipients in Vax004, all of whom were uninfected at month 12.5. Additional plasma samples were obtained from 169 chronically infected individuals who were not part of any vaccine clinical trial and who were antiretroviral drug-naïve; these individuals were infected with HIV-1 subtypes A (n=8), B (n=59), C (n=57), D (n=8), CRF01-AE (n=13), CFR02_AG (n=2), CRF07_BC (n=1), CRF10_CD (n=2), AC (n=4), AD (n= 3) and ABCD (n=2). HIV-1 genetic subtypes were determined by single genome amplification and sequencing of a single plasma gp160 gene as described [40,41]. All clinical trials were conducted in accordance with the Declaration of Helsinki and local institutional review board requirements. Written informed consent was obtained from all clinical trial subjects.

### Design of overlapping peptides

Peptides were designed to cover the entire gp160 consensus sequences for HIV-1 Group M, subtypes A, B, C, D, CRF01_AE and CRF02_AG [19] for a total of 1423 peptides (15-mers overlapping by 12 amino acids). Peptide sequences were generated by alignment of the 7 consensus gp160 sequences using the LANL PeptGen tool (www.hiv.lanl.gov), so that peptides remain in register throughout the Env despite insertions and deletions, and identical peptides found in more than one subtype were only represented once. A listing of all of the peptides and their sequences may be found in Table S1.

### Peptide synthesis and microarray printing

PepStar peptide microarrays were produced by JPT Peptide Technologies GmbH (Berlin, Germany). A total of 1423 tiled Env peptides (peptide length 15 aa) were synthesized on cellulose membranes using SPOT synthesis technology. After a final synthesis step attaching a reactivity tag to each peptide’s N-terminus, the side chains were deprotected and the solid-phase bound peptides were transferred into 96-well microtiter filtration plates (Millipore, Bedford, MA, USA). Subsequently the peptides were treated them with aqueous triethylamine [2.5% (v/v)] cleaving the peptides from the cellulose membrane. The peptide-containing solution was centrifuge-filtered into daughter plates and the solvent was removed by evaporation under reduced pressure. Quality control measurements using LCMS were performed on random samples of final library. Dry peptide derivatives (50 nmol) were dissolved in 35 µl of printing buffer and transferred into 384-well microtiter plates. Peptide microarrays were produced using high performance contact printers on epoxy-modified slides (PolyAn; Germany). All peptides and controls were deposited in three identical subarrays, enabling analysis of assay homogeneity and reliability of the results. Peptide microarrays were scanned after printing process for identification and quality control of each individual spot. Subsequently, peptide microarray surfaces were deactivated using quenching solutions, washed with water and dried using microarray centrifuges. Resulting peptide microarrays were stored at 4°C until use.

### Peptide array binding assay

Microarray binding was performed using the HS4800 Pro Hybridization Station (Tecan, Männedorf, Switzerland). All arrays were blocked with Superblock T20 PBS blocking buffer for 0.5 hour at 30°C, followed by a 2 hr incubation at 30°C with heat inactivated plasma diluted 1:100 in Superblock T20. Arrays were incubated for 45 minutes at 30°C with anti-IgG Cy5 secondary antibody (1.5 µg/ml final concentration) diluted with Superblock T20. Washes between all steps were with PBS containing 0.1% Tween. Arrays were scanned at a wavelength of 635 nm using an Axon Genepix 4300 Scanner (Molecular Devices, Sunnyvale, CA, USA) at a PMT setting of 600, 50% laser power. Images were analyzed using Genepix Pro 7 software (Molecular Devices).

### Data Analysis

#### Data pre-processing and normalization

Foreground and background intensities from peptide microarray scans were loaded from GenePix image (gpr) files. Background-corrected intensities were estimated using the normexp method, developed and reviewed by Ritchie et al. [42], implemented in the limma R package [43], where within-slide peptide replicates were summarized by their median. Resulting peptide intensities were log_2_ transformed and corrected for peptide sequence composition biases using a custom normalization linear model [44]. Normalized values for each peptide were corrected for baseline by subtracting the corresponding pre-vaccination intensities. Due to a lack of pre-infection samples, the HIV-1-positive data were baseline corrected by subtracting, for each peptide, the mean intensity of the 10 HIV-1-negative samples.

#### Data smoothing and positivity calls

Normalization methods help remove systematic biases, but experimental and technical variation may remain leading to background noise. Normalized (and baseline corrected) intensities alone also fail to take advantage of the overlapping nature of peptides on the array where we expect that the binding effects of two overlapping peptides will be positively correlated. Therefore normalized peptide intensities were smoothed using a sliding window of 9 amino-acids to borrow strength across neighboring peptides and to reduce signal variability when calling positive peptides. This smoothing step can be made genetic subtype specific, or made across all genetic subtypes, to borrow strength across multiple peptides. Aggregate and subtype specific frequency of responses were computed across all individuals within a dataset by dichotomizing smoothed peptide intensities using a log_2_ fold-change threshold of 1.1. This threshold was estimated by using the results obtained with plasma from 20 placebo recipients in RV144, resulting in an estimated false discovery rate that was less than 10%.

Major reactive regions in gp160 were identified by estimating the aggregate frequency of responses for all test samples within a study group. Peptide sequences centered at the maximum response within each major reactive region were used as candidate variables for correlates of risk analysis. Aggregate variables also were defined by averaging, for a given major reactive region, all individual subtype-specific candidate variables. This resulted in 20 subtype specific variables and 4 aggregate variables that were assessed for correlates.

#### Correlates of risk

All immune variables identified here by peptide array binding, in addition to the six primary variables defined previously [32], were assessed as correlates of infection risk (CoR) by using the statistical methods specified in the original correlates study [32]. Briefly, for each immune biomarker, logistic regression accounting for the sampling design was used to estimate the odds ratio (OR) of infection, controlling for gender and baseline behavioral risk. In addition, based on the idea that high levels of Env-specific IgA antibodies may have mitigated the effects of protective antibodies [32] we included univariate-CoR results based on a model that also controls for IgA level. All calculations were performed by using the osDesign R package [45]. To facilitate comparison of estimated ORs, all variables were standardized to have mean=0 and standard deviation=1. Interaction effects between any two given variables were tested using the same logistic regression framework including the two variables and their interaction. Resulting p-values were corrected for multiple testing using a False Discovery Rate (FDR) approach to define q-values [46,47]. The q-value is the minimal false discovery rate at which a statistical test result may be called significant. For example, using a q-value of 0.2 would mean that up to 20% of the declared discoveries could be false positive. Q-values are optimized for exploratory discoveries at the expense of an acceptable risk of false positive results.

#### Properties of gp120 core structures

All gp120 core PDB structure files (1G9M) [48], 1RZK [49], 2B4C [50], 2NY7 [51], 3JWD [52], 3JWO [52], and 3LQA [53] were prepared at pH=7 using the PDB 2PQR framework [54] with protonation states for all residues determined using PROPKA3.0 [55] program. Electrostatic surface potential (ESP) calculations were performed by numerically solving the full nonlinear form of the Poisson-Boltzmann equation using the APBS software v1.4 [56] at a temperature of 310K with standard parameters. ESP grid sizes and granularities were determined using the psize.py script supplied with the APBS software. Partial charges and van der Waals parameters were taken from the AMBER 99 force field [57]. The solvent accessible surface area (SASA) of all structures was determined using the “measure” function of VMD v1.9.1 [58] using a 0.14nm radius, and restricting the resulting surface to only include the residues within reactive peptide regions. The mean electrostatic surface potential (MESP) was calculated by linearly interpolating between all immediately neighboring ESP grid values along the SASA, summing across all SASA grid locations, and dividing by the total surface area. Secondary structure assignment was performed using the DSSP software package [59]. All residues were assigned a single-letter code, each pertaining to the different secondary structure classes assigned by DSSP: A – alpha helix, B – isolated beta bridge (beta strand), E – extended beta strand (beta sheet), G -3/10 helix (3 helix), I – pi helix (5 helix), T – hydrogen bonded turn (helix-like), S – bend (strand-like), X – unclassified (unknown or “coil”). A similar approach was used to account for properties of antibody-bound V2 reactive regions from three different structures (3U4E, 4HPO and 4HPY) [12,36].

## Results

### Magnitude and frequency of IgG binding in peptide arrays

**Figure 1:**
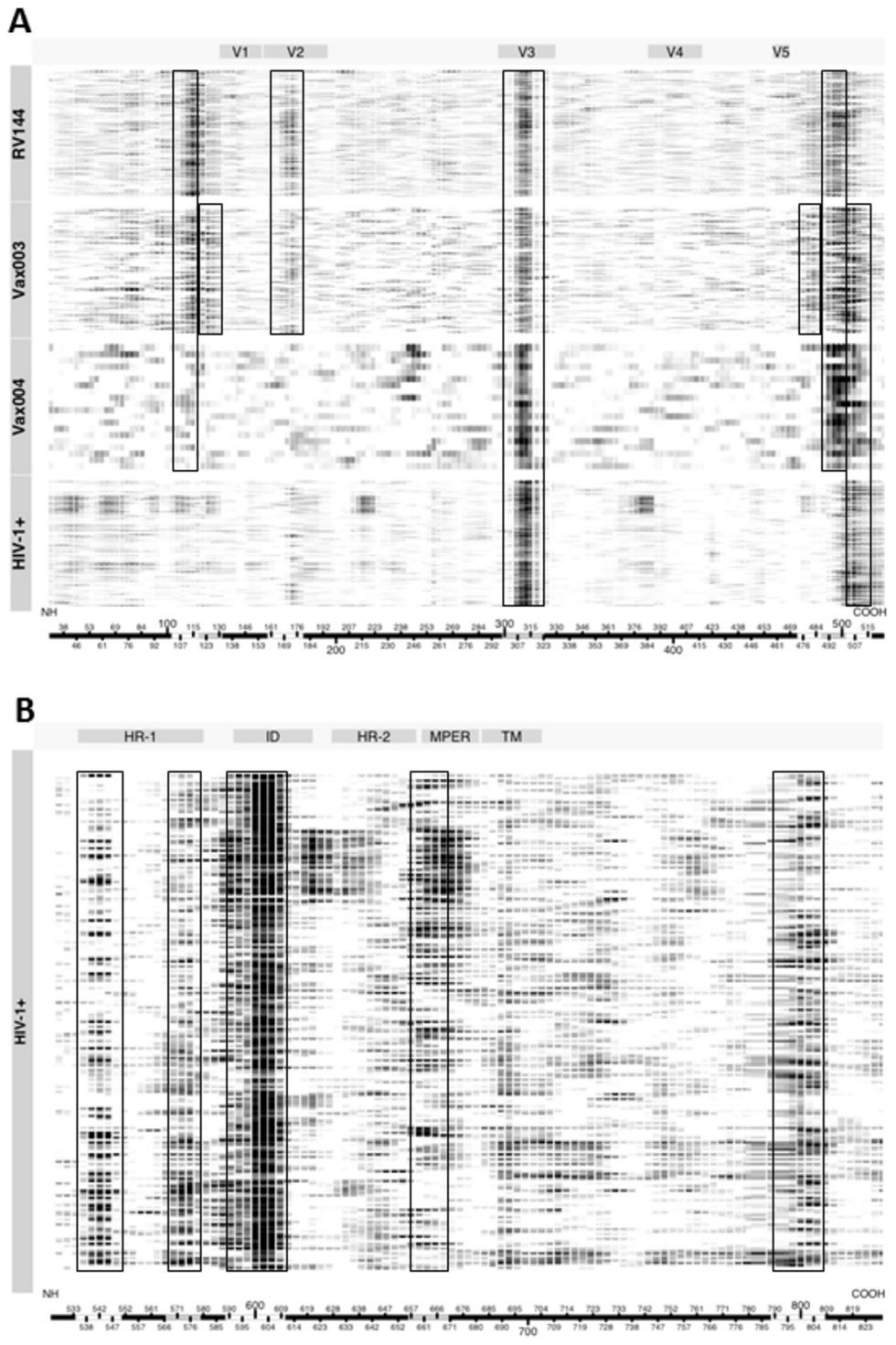
Heatmaps of smoothed normalized peptide binding values for samples from RV144, Vax003, Vax004 and HIV-1-infected individuals as a function of HxB2 coordinates. Each row represents a sample from a single individual, where stronger intensities of binding are shown as darker images. Columns represent amino acid positions using HxB2 numbering from the amino terminus (NH) to the carboxy terminus (COOH) as shown along the x-axis of each heatmap. Within the x-axis bar, areas of white and gray are used to show regions of strongest reactivity. Boxes are used to show group-specific regions of strongest reactivity. A. Gp120 peptides. B. Gp41 peptides.

Env-specific plasma IgG was assessed with overlapping peptides (15 mers overlapping by 12) spanning the entire consensus gp160 of HIV-1 subtypes A, B, C, D, CRF01_AE, CRF01_AG and Con-M. Figure 1A shows a heatmap of aggregate smoothed binding intensities against the gp120 peptides for samples obtained at peak immunity (2 weeks post 4^th^ inoculation) from 246 vaccine recipients in RV144, 90 vaccine recipients in Vax003 and 20 vaccine recipients in Vax004. Also shown are binding intensities for a multi-subtype panel of plasma samples from chronically HIV-1-infected individuals. Several linear B cell epitopes were identified. Among them, the V3 loop and C-terminus of the C5 region of gp120 were major targets for the IgG response in all four groups of subjects. C5 contained three adjacent reactive regions, designated C5a, C5b and C5c. C5a was a dominant response in Vax003, whereas C5b was a dominant response in all groups except for HIV-1-infected individuals. C5c was a dominant response in all groups except for RV144. The C1 region of gp120 was another major response in RV144, Vax003 and Vax004 but not in HIV-1-infected subjects. C1 contained two adjacent reactive regions, designated C1a and C1b, where C1a was a major response in RV144, Vax003 and Vax004, while the C1b response was primarily seen in Vax003. Finally, the V2 region of gp120 was a major response in RV144 and Vax003 but not in Vax004 and HIV-1-infected individuals. Overall, vaccination induced responses to more epitopes than did HIV-1 infection, including a V2 response that was substantially stronger in RV144 and Vax003 (including CRF01_AE infected subjects) and was absent in Vax004.

**Figure 2:**
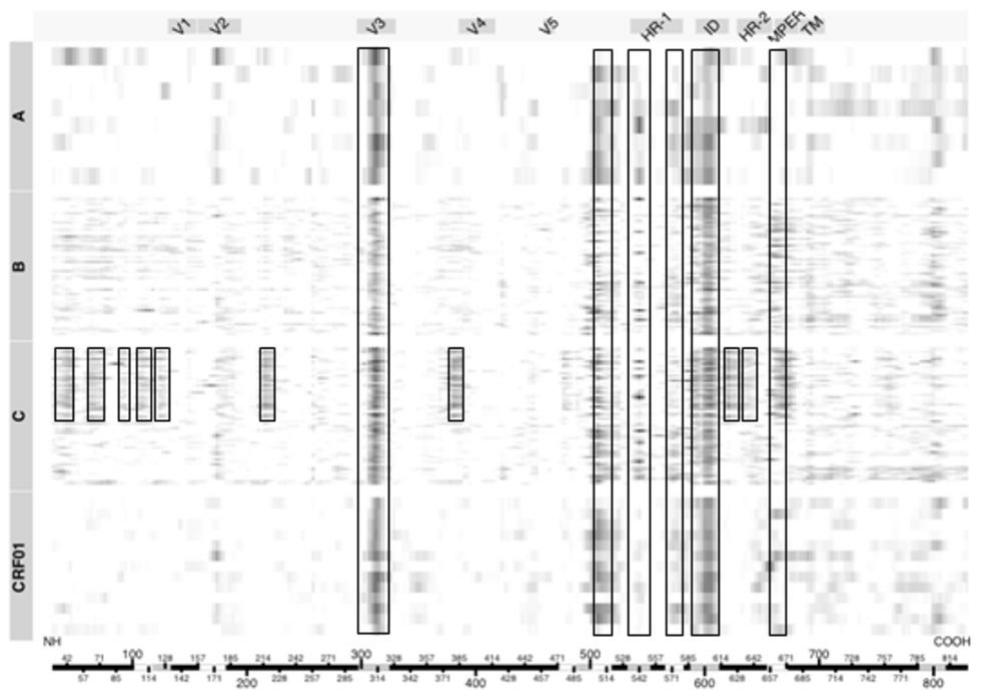
Heatmap of smoothed normalized gp160 peptide binding values for samples from HIV-1-infected individuals as a function of genetic subtype and HxB2 coordinates. Each row represents a sample from a single individual, where stronger intensities of binding are shown as darker images. Columns represent amino acid positions using HxB2 numbering from the amino terminus (NH) to the carboxy terminus (COOH) as shown along the x-axis. Within the x-axis bar, areas of white and gray are used to show regions of strongest reactivity from Figure 1. Boxes are used to show the regions of strongest reactivity from Figure 1, plus additional regions of interest.

Additional major responses in HIV-1-infected individuals targeted several regions in gp41, including the N- and C- termini of heptad repeat 1 (HR-1), the immunodominant domain (ID) and the membrane proximal external region (MPER) (Figure 1B) (gp41 reactivity was not expected in the vaccine recipients because the immunogen was gp120). A subset of individuals infected with subtype C HIV-1, selected among 96 individuals for having the greatest neutralizing activity against a panel of six Tier 2 clade C viruses, demonstrated additional binding specificities. Thus, in addition to V3, C5, HR-1, ID and MPER, these subjects frequently responded to epitopes in the C1 (including C1a and C1b), C2, and C3/V4 regions of gp120, plus two sites in the HR-2 region of gp41 (Figure 2). This pattern was not associated with neutralization potency in general among the entire dataset, suggesting it is a relatively unique feature of stronger responses in subtype C-infected subjects. Figure 2 also shows that the MPER responses were mostly seen in subjects who were infected with subtype B and C viruses, with only rare reactivity in subtype A-infected subjects.

**Figure 3:**
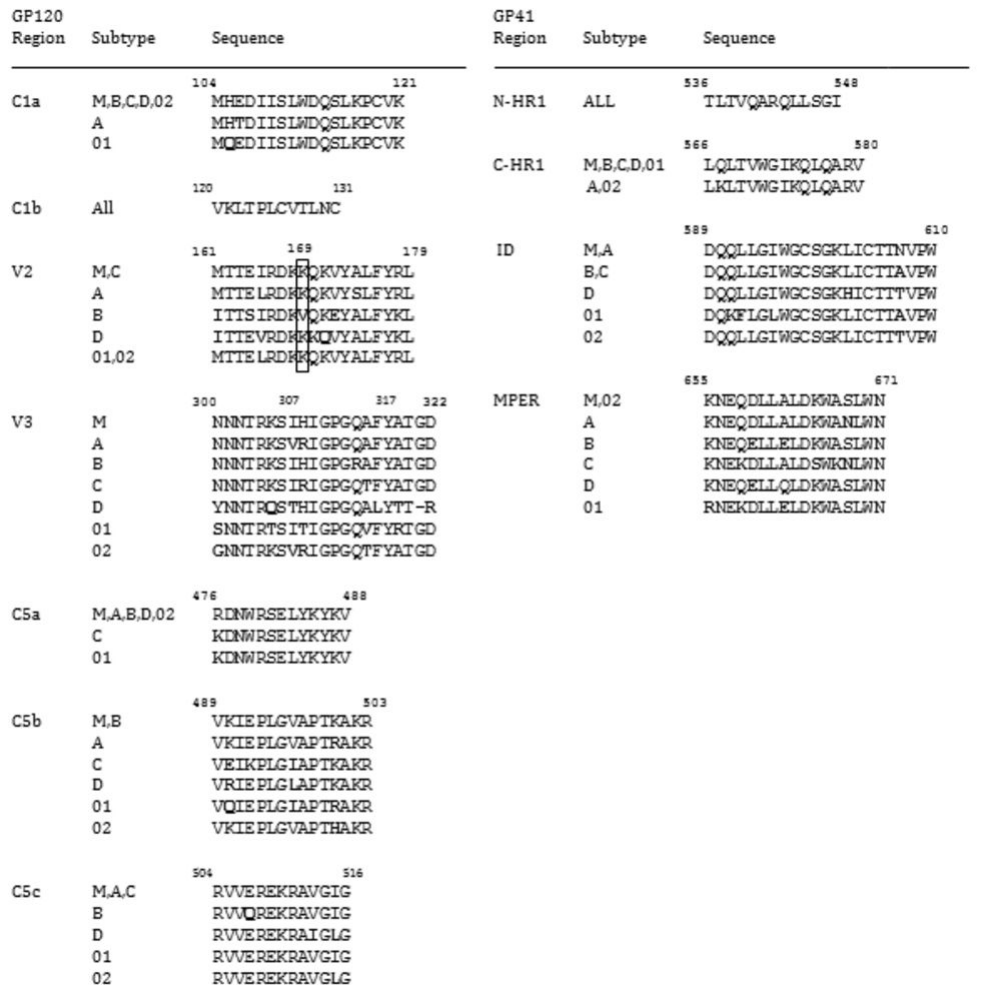
IgG response rates by peptide genetic subtype. Response rates (percent positive responses) are shown for major reactive regions in gp120, as color-coded in the legend.

The gp41-ID reactive region was 23 amino acids in length, contained a disulfide bridge, and was relatively conserved among HIV-1 subtypes except at position 607, which accommodated asparagine, alanine or threonine; subtypes D and CRF01_AE contained additional changes, most notably in CRF01_AE (Figure 3). The two dominant epitopes in HR1 were highly conserved, with only a single change at position 567 in C-HR1 of subtype A and CRF02_AG. The MPER reactive region was the most variable and contained the 2F5 epitope but did not contain the most membrane proximal residues for other MPER-specific bnAbs.

**Figure 4:**
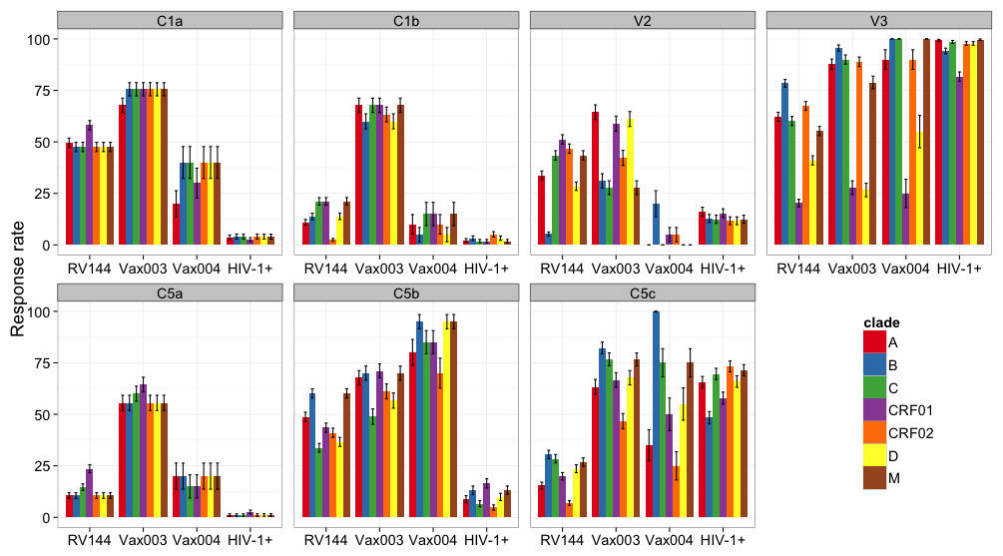
Sequences of major reactive regions in gp120 and gp41. Sequences are shown for individual genetic subtypes in the major IgG binding regions of gp120 and gp41. Borders were defined by overlapping peptide binding intensities (only reactive regions are shown). Amino acid residues that differ from the group M consensus are shown in boldface type. Boxed is a key position in V2 that was identified by genetic sieve analyses of breakthrough viruses in RV144. The HXB2 numbering system is used to identify amino acid sites.

IgG responses were compared based on frequencies of binding to different genetic subtype of the gp120 peptides (Figure 4). The overall response rate to C1a was highest in Vax003 (58-74%), followed by RV144 (48-61%) and Vax004 (24-35%). C1a spans a highly conserved region, and the consensus sequences for the M group, B, C, D, and CRF02_AG are identical in this region (Figure 3). There is only a single amino acid change in subtype A and one in CRF01_AE (Figure 3). These changes resulted in a somewhat higher frequency of responses in RV144, and diminished responses in Vax004, to subtype A and CRF01_AE relative to the other C1a subtypes. The peptide that is associated with C1b reactivity is so highly conserved that it is identical in all subtypes (Figure 3). C1b reactivity was only seen in Vax003 and as expected, it was high for all subtypes (60-68% response rates) (Figure 4).

The response rate to V2 was substantially higher in Vax003 and RV144 than in Vax004 and HIV-1-infected subjects (Figure 4). The anti-V2 response in RV144 was highly cross-reactive, with reactivity greatest against CRF01_AE and CRF02_AG consensus V2 peptides (which are identical) followed by Con-M and subtype C consensus V2 peptides (which are identical) and subtype A V2 peptides (38-55% response rates). Less frequent reactivity was seen to the consensus of subtypes D and B (27% and 8% response rate, respectively), which were very distinct in the V2 peptide region (Figure 3). The response in Vax003 was somewhat higher overall and reacted with subtypes A, D and CRF01_AE V2 peptides, in this descending order (60-72% response rates), with less frequent reactivity to CRF02_AG and subtypes B, C and group M V2 peptides (37-50% response rates). It is possible that the CRF01_AE gp120 presented the V2 loop more favorably than the subtype B gp120s, driving a more intense V2-CRF01_AE peptide response.

Notably, the response rate to V2 CRF01_AE in RV144 (52%) was no higher than in the non-protective Vax003 trial (61%). The reactive region in V2 contained position 169 that demonstrated a sieve effect in RV144 [35] and that is critical for the binding of V2-specific Abs from RV144 [33,34], including monoclonal Abs that mediate ADCC activity [36]. Among the reactive V2 peptides, subtype B was the only one lacking K169 (Figure 3), and it was the least reactive with RV144 and Vax003 samples (Figure 4). As noted previously [33], the linear epitope represented in these reactive V2 peptides is located proximal to, but does not span the LDI/V motif that has been shown to mediate gp120 binding to the α4β7 integrin [60].

For the most part, all four groups of subjects showed high response rates across multiple genetic subtypes of the V3 peptides (Figure 4) despite considerable sequence variability within the reactive region of 21 amino acids (Figure 3). An exception was a diminished response to CRF01_AE and subtype D in the three vaccine trials. The relatively low response rates against V3 CRF01_AE in RV144 (25%) and Vax003 (37%) were unexpected because both vaccines were comprised in part of two CRF01_AE gp120 immunogens. The V3 CRF01_AE consensus peptides were clearly reactive because a 100% response rate was seen with samples from HIV-1-infected subjects. Thus, for reasons that are not understood, the CRF01_AE gp120 immunogens elicited stronger IgG responses against non-CRF01_AE V3 peptides than against subtype-matched V3 peptides. Of note, the strongest vaccine-elicited V3 responses were to subtype B consensus peptides, and there was a B subtype gp120 in addition to two CRF01_AE gp120s in both RV144 and Vax003. It is possible that the B subtype gp120 presented the V3 loop more favorably than the CRF01_AE gp120s, driving a more intense V3 subtype B peptide response [34].

A high response rate to C5a was seen only in Vax003 (56-64% response rate) (Figure 4). C5a is a highly conserved peptide (Figure 3), and the response was naturally conserved across all subtypes (Figure 4). Response rates to C5b were highest in Vax004 (70-95%), followed by Vax003 (49-71%) and RV144 (34-60%), with only negligible responses seen in HIV-1-infected subjects (<20%). Minor differences were seen in the response across subtypes, which might be explained by a moderate level of sequence variability in C5b (Figure 3). Response rates to C5c were relatively high in Vax003, Vax004 and HIV-1-infected subjects (25-100%), and were low in RV144 (7-30%). As expected from the low sequence variability in C5c, responses to these peptides were relatively conserved across subtypes. One notable exception was a diminished response against CRF02_AG relative to the other subtypes in all three vaccine trials.

### Structures of the major B cell epitopes

**Figure 5:**
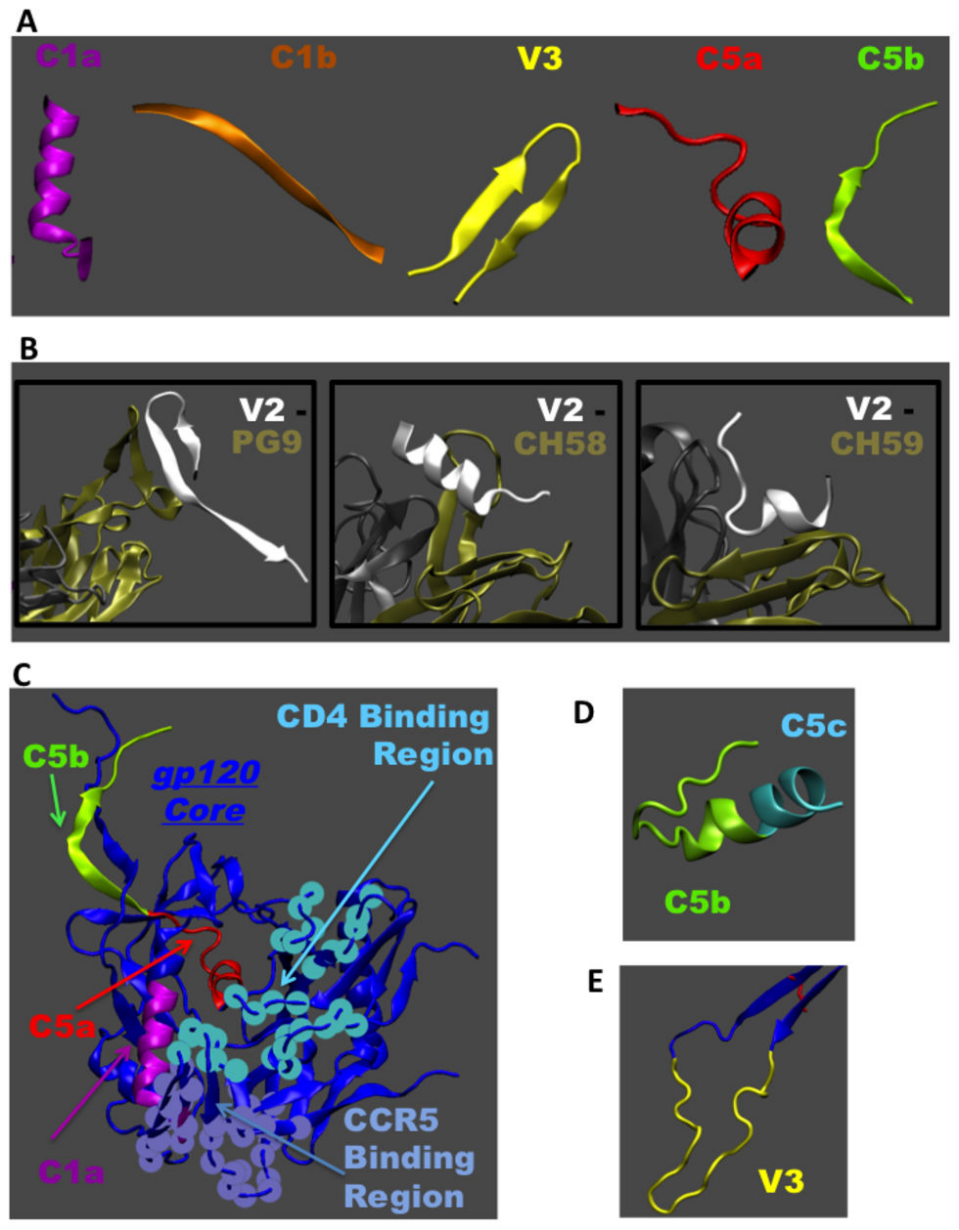
Known conformations of reactive peptides. **A**. Conformations of reactive peptides known from solved X-ray structures of gp120 core or fragments of gp120 in complex with antibodies. **B**. V2 peptide is shown with respect to different conformations it adopts depending on the bound antibody. Structures with antibodies PG9 (PDB: 3U4E), CH 58 (PDB: 4HPO) and CH 59 (PDB: 4HPY) are shown where tan and gray colors indicate the light and heavy chains of the antibody, respectively. **C**. The CD4 bound structure of gp120 with N-and C- terminal regions is used as a template to show the C1a, C5a, C5b peptides in the context of entire gp120 monomer structure (PDB: 3JWD). Also shown are the CD4 and CCR5 binding regions respect to these peptides. **D**. NMR structure of isolated region of C-terminal end of gp120 showing C5b and part of C5c peptides (PDB: 1MEQ). **E**. V3 peptide is shown with respect to the rest of the gp120 core (PDB: 2B4C).

Next we evaluated conformational and other physical properties of the major reactive peptide regions in gp120, as the structures of these peptide regions in the context of the gp120 protein may reveal what makes them antigenic. Table 1 shows that all of the reactive regions are predicted to be solvent exposed. Figure 5 shows the conformations of these reactive regions from known structures of the gp120 core or fragments of gp120 solved with bound antibodies. C1a adopt a mostly helical conformation, whereas C5a forms a partially helical conformation (Figure 5A). C1b and C5b mostly exist as a β beta strand structure (Figure 5A). Figure 5B shows that V2, which is from a flexible region of gp120, adopts multiple structures upon binding to antibodies, including β strand, α helix and coil structures [12,36].

**Table 1:** Structural and physical properties of reactive peptide regions in the context of known crystal structures of liganded gp120.

		**Properties^1^**									
**PDB**	**Sequence**	**SASA^2^**	**MESP^2^**	**%H**	**%B**	**%E**	**%G**	**%I**	**%T**	**%S**	**%X**
**C1a Region:**											
1G9M	MHEDIISLWDQSLKPCVK	714	-5.9	55.6	0.0	11.1	0.0	0.0	11.1	11.1	11.1
1RZK	MHEDIISLWDQSLKPCVK	649	-22.7	38.9	0.0	11.1	0.0	0.0	27.8	5.6	16.7
2B4C	MQEDIISLWDQSLKPCVK	784	-25.6	55.6	0.0	11.1	0.0	0.0	11.1	5.6	16.7
2NY7	MHEDICSLWDQSLKPCV	1406	-19.3	0.0	0.0	17.6	17.6	0.0	0.0	5.9	58.8
3JWD	MHEDIISLWDQSLKPCVK	563	-7.9	61.1	0.0	11.1	0.0	0.0	0.0	16.7	11.1
3JW0	MHEDIISLWDQSLKPCVK	622	-9.8	61.1	0.0	11.1	0.0	0.0	0.0	16.7	11.1
3LQA	MHQDIISLWDQSLKPCVK	679	-18.6	66.7	0.0	11.1	0.0	0.0	0.0	11.1	11.1
**C1b Region:**											
1G9M	VKLTPLCV	509	12.1	0.0	0.0	50.0	0.0	0.0	0.0	0.0	50.0
1RZK	VKLTPLCV	505	-2.3	0.0	0.0	87.5	0.0	0.0	0.0	0.0	12.5
2B4C	VKLTPLCV	526	-13.3	0.0	0.0	75.0	0.0	0.0	0.0	0.0	25.0
**V2 Region:**											
3U4E	ITTELRDKKQKAYALFYR	508	-0.4	0.0	0.0	77.8	0.0	0.0	16.7	0.0	5.6
4HPO	DKKQKVHALFYKL	1168	5.8	69.2	0.0	0.0	0.0	0.0	0.0	0.0	30.8
4HPY	KKQKVHALFYK	1085	8.4	0.0	0.0	27.3	0.0	0.0	27.3	0.0	45.5
**V3 Region:**											
2B4C	NQNTRKSIHIGPGRAFYTTGE	2515	25.0	0.0	0.0	19.0	0.0	0.0	19.0	9.5	52.4
**C5a Region:**											
1G9M	RDNWRSELYKYKV	210	9.3	61.5	0.0	23.1	0.0	0.0	15.4	0.0	0.0
1RZK	RDNWRSELYKYKV	259	-0.4	61.5	0.0	23.1	0.0	0.0	15.4	0.0	0.0
2B4C	RDNWRSELYKYKV	320	-7.1	38.5	0.0	23.1	0.0	0.0	30.8	7.7	0.0
2NY7	RDNWRSELYKYKV	176	-10.4	30.8	0.0	23.1	0.0	0.0	38.5	0.0	7.7
3JWD	RDNWRSELYKYKV	157	-22.5	38.5	0.0	23.1	0.0	0.0	30.8	7.7	0.0
3JW0	RDNWRSELYKYKV	153	-27.8	38.5	0.0	23.1	0.0	0.0	30.8	7.7	0.0
3LQA	KDNWRSELYKYKV	240	22.4	61.5	0.0	15.4	0.0	0.0	0.0	15.4	7.7
**C5b Region:**											
3JWD	VKIEPLGVAPTKA	1328	-2.6	0.0	0.0	69.2	0.0	0.0	0.0	0.0	30.8
3JW0	VKIEPLGVA	740	-10.6	0.0	0.0	88.9	0.0	0.0	0.0	0.0	11.1

^1^ SASA, Solvent accessible surface area; MESP, Mean electrostatic surface potential at neutral pH; %H, Percent alpha helix; %B, Percent isolated bate bridge (beta strand); %E, Percent extended beta strand (beta sheet); %G, Percent 3/10 helix (3 helix); %I, Percent Pi helix (5 helix); %T, Percent hydrogen bonded turn; %S, Percent bend; %X, Percent unclassified (unknown or coil).

^2^ For the V2 regions, which used liganded structures, we used SASA-B (solvent accessible surface area of peptide that is buried by antibody) and MESP-E (mean electrostatic surface potential at neutral pH for the region making contact with antibody).

It is also useful to know whether the peptides are capable of adopting the same conformation when fragmented from the remainder of the gp120 structure. The ability of an isolated fragment to adopt the same conformation as when it is part of gp120 protein may further confirm the antigenicity of that region. At the same time, if the fragmented peptides adopt different structures or become random coils, then it raises the question of why they are reactive. Though the structure of the native gp120 trimer is not known, several three-dimensional structures have been reported for the liganded conformation of gp120 monomers. Most of these monomer structures capture only the gp120 core, which omits the V1, V2 and V3 loops and N- and C-termini. According to a recent study, when the V1V2 domain and gp41 contacts are removed, the native monomer gp120 core adopts a conformation similar to a CD4 bound monomer conformation [61]. In Table 1, we have listed several properties of these reactive peptide regions based on known X-ray structures of liganded gp120 [48-53]. Trends in properties, such as secondary structure and solvent exposure, are consistent among different peptide within a reactive region. A notable exception is the b12-bound structure of C1a(2NY7), which was more solvent exposed, lacked alpha helical structure and contained beta sheet and 3-helix conformations not seen in other representatives of this region.

The C1a and C1b peptides occur in the inner domain of the gp120 core. C1b forms the stem of the V1V2 loop projection at the apex of gp120. Only the C1a peptide occurs in the outer domain of the gp120 core. For each gp120-gp41 heterodimer, the N- and C- termini of gp120 come spatially close together and form the interface to gp41. These regions are conformationally variable and can be influenced by proteins that pack nearby. Recently, a liganded gp120 core structure was solved with N- and C-termini, but lacking the gp41 protein that interacts with this region [52]. We used that structure to highlight the reactive regions corresponding to C1a, C5a and C5b in the context of the gp120 structure (Figure 5C). These regions are not part of the CD4 or CCR5 binding regions. The C1a region is helical in most of the liganded gp120 core structures. However, this region may not be helical in its native conformation, as the inner domain undergoes significant conformational changes. As mentioned above, this region is non-helical in the b12-bound structure of gp120 [51]. In all known structures, C5a is helical (Table 1). It is not known whether the C5a peptide, when fragmented from rest of the gp120 core, adopts a helical structure. However, it appears that the C5b and C5c peptides can adopt different conformations when fragmented from gp120. Mapping of the epitope recognized by human monoclonal Ab 1331A places it in the C5b region, and molecular modeling results suggest it to be a discontinuous epitope in which the amino acids critical for Ab binding are located on opposite sides of the hydrophobic pocket, which is thought to be of importance for the interaction of HIV-gp120 with gp41 [62]. Figure 5D shows the conformation of C5b and part of C5c determined through NMR in a lipid environment [63]. In this case, the fragment is helical, not β stranded as part of gp120. Thus, the C-terminal region, where C5b and C5c are located, can adopt different conformations depending on its environment.

Peptides from variable regions are expected to be flexible in their native environment. Depending on the antibodies that bind to that region or the context of the rest of the gp120 structure, these flexible regions can adopt different conformations. As mentioned above, the reactive V2 peptide region can adopt multiple structures, including a β sheet conformation when bound to PG9 (Figure 5B). The same peptide region takes on helical conformation when bound to antibody CH 58, and it is predominantly random coil when bound to antibody CH 59. This conformational plasticity of V2 peptides is indicative of this region being intrinsically disordered and its conformation being strongly influenced by the binding partners and environment. The conformational plasticity coupled with sequence variation enables the V2 peptide to accommodate multiple binding modes with antibodies. As shown in Table 1, the amount of surface area that takes part in V2 peptide-antibody complex as well as the nature of electrostatic surface potential of V2 seen by the antibodies are similar for CH 58 and CH 59 but different to that of PG9. Even though this region may be disordered in the gp120 monomer, the conformational variability of this region in a gp120 trimer is expected to be restricted due to interaction with neighboring monomers. The reactive V3 peptide region at the crown of the V3 loop (Figure 5E) is also quite flexible. This region takes on β strand–turn–β strand conformation when bound to many anti-V3 monoclonal Abs [64]. Again, it was only possible to determine the structure of V3 peptide when bound to an antibody, indicating that it may be flexible within the gp120 monomer in the absence of antibody. Similar to V2 peptides, V3 is thought to assume a more stable conformation in the context of the native Env trimer.

### V2 peptide-specific IgG as a correlate of infection risk in RV144

Among the 17 different types of immune assays and their 152 component variables used to assess correlates of infection risk in RV144, 6 assays were chosen as primary variables for optimal statistical power when adjusting for multiple comparisons [32]. The primary variables included Env-specific plasma IgA, Env-specific plasma IgG binding avidity, gp70-V1V2-specific plasma IgG, neutralizing Abs, ADCC and Env-specific CD4^+^ T cells. The major findings (based on a multivariate model including all six primary variables) were that gp70-V1V2-specific plasma IgG inversely correlated with infection rate (odds ratio 0.57, P=0.03, q=0.08) and that Env-specific plasma IgA was a direct correlate of infection rate (odds ratio 1.54, P=0.03, q=0.08) [32]. The remaining 4 variables showed inverse correlations with infection rate in subjects who had low levels of Env-specific plasma IgA. Also, there was no evidence that the gp70-V1V2 and IgA variables interacted; thus they were independent correlates if risk.

Although IgG responses to C1, V2, V3 and C5 in peptide arrays were part of the primary RV144 correlates study, only aggregate responses across all genetic subtypes within each of these regions were analyzed, and individual subtypes were not considered. In the primary study, IgG responses to all V2 subtypes in aggregate showed a borderline significant inverse correlation with infection risk, whereas no significant association with risk was seen for the C1, V3 and C5 responses [32]. In addition, no interaction analyses were tested between the peptide array data and the six primary variables defined in the main correlates study [32].

**Figure 6:**
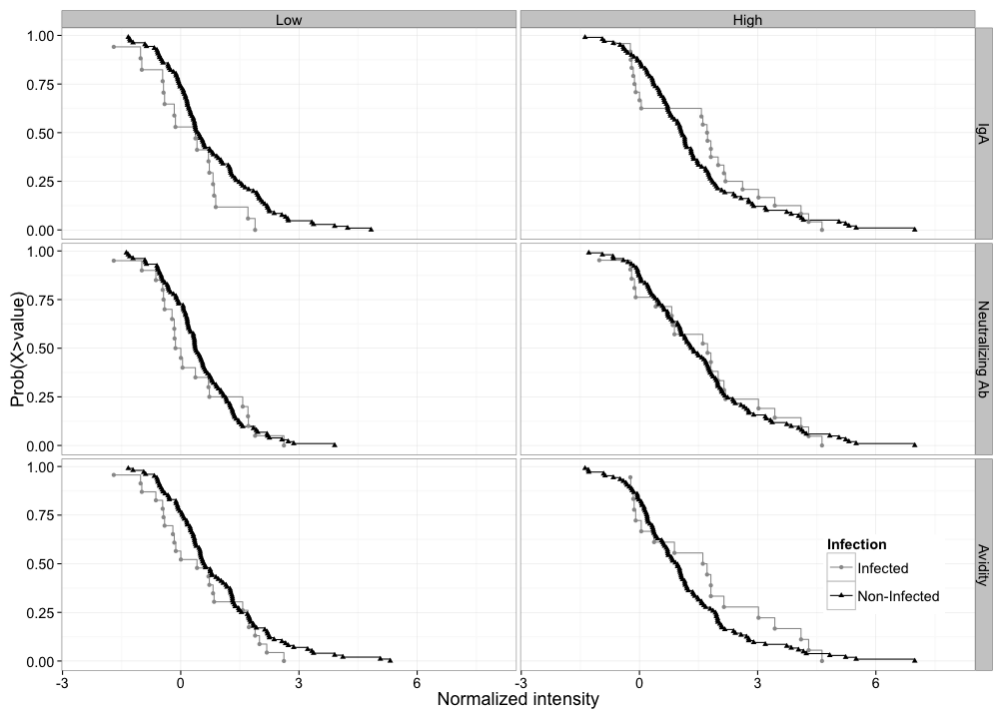
Subtype-specific and aggregate V2 responses in RV144. Boxplots are stratified by infection status for peptides centered at the maximum hotspot region (position 174). Individuals with different risk and gender categories (used in our correlate model) are shown with different symbols and grey shades.

We assessed the IgG responses by individual genetic subtype and by aggregate subtypes within each major reactive region of gp120 as correlates of infection risk in RV144. Because the large number of comparisons diminished the robustness of our analysis, we included the 6 primary immune variables and subjected them to the same corrections for multiple tests as a reference. Figure 6 shows the distribution of aggregate V2 and subtype-specific V2 responses in infected and non-infected vaccine recipients. Stronger V2 responses correlated with a lower infection risk in the case of responses to V2.1 (OR=0.62, p=0.014, q=0.24), followed by V2.MC2 (OR=0.66, p=0.027, q=0.24), V2.aggregate (OR= 0.65, p=0.030, q=0.24), and V2.D (OR=0.66, p=0.037, q=0.24) (Table 2). Also, a trend was seen for the response to V2.A (OR=0.70, p=0.060, q=0.24). All five correlations were at least as significant as the correlation seen with the primary gp70-V1V2 variable as assessed by the same univariate model (OR= 0.71, p=0.063, q=0.24). No significant correlation was seen for IgG responses to V2.B, or any subtype of the C1, V3 and C5 peptides, although higher IgG to C5b.A showed a trend toward an increased risk of infection (OR=1.40, p=0.052, q=0.24) (Table 2). After adjusting for IgA level (Table 2), identified correlates became even more significant. Thus, all V2 correlates except V2.B now had q-values <0.15, and the IgA-adjusted V2.1 correlate (OR=0.54, p=0.004, q=0.12) remained more significant than the IgA adjusted gp70-V1V2 correlate (OR=0.63, p=0.02, q=0.13). These results indicate that stronger linear V2-specific IgG responses, especially against V2 CRF01_AE, were associated with a lower risk of HIV-1 infection in RV144.

**Table 2:** Peptide-specific IgG responses as correlates of a lower infection risk in RV144.

	**Infection risk**				
**IgG Variable^1^**	**Odds Ratio^2^**	**CI lower^3^**	**CI upper^3^**	**p-value^4^**	**q-value^5^**
C1a.aggregate	1.08 (0.97)	0.76 (0.68)	1.51 (1.38)	0.68 (0.85)	0.75 (0.86)
V2.aggregate	**0.65 (0.59)**	**0.44 (0.39)**	**0.96 (0.90)**	**0.030 (0.013)**	**0.24 (0.13)**
V3.aggregate	0.81 (0.78)	0.57 (0.53)	1.17 (1.13)	0.27 (0.19)	0.58 (0.51)
C5.aggregate	1.24 (1.23)	0.88 (0.87)	1.73 (1.73)	0.22 (0.24)	0.53 (0.54)
C1.1	1.08 (1.03)	0.76 (0.68)	1.51 (1.38)	0.68 (0.85)	0.75 (0.86)
C1.MABCD2	1.16 (0.92)	0.82 (0.74)	1.63 (1.51)	0.40 (0.75)	0.65 (0.86)
V2.1	**0.62 (0.54)**	**0.42 (0.36)**	**0.91 (0.82)**	**0.014 (0.0042)**	**0.24 (0.12)**
V2.A	0.70 (0.65)	0.48 (0.44)	1.02 (0.96)	0.060 (0.031)	0.24 (0.15)
V2.B	0.91 (0.89)	0.64 (0.64)	1.29 (1.26)	0.59 (0.52)	0.73 (0.68)
V2.D	**0.66 (0.63)**	**0.44 (0.42)**	**0.98 (0.94)**	**0.037 (0.025)**	**0.24 (0.14)**
V2.MC2	**0.66 (0.60)**	**0.44 (0.40)**	**0.95 (0.91)**	**0.027 (0.016)**	**0.24 (0.13)**
V3.1	0.86 (0.84)	0.60 (0.58)	1.23 (1.21)	0.41 (0.34)	0.65 (0.58)
V3.2	0.83 (0.80)	0.58 (0.55)	1.19 (1.15)	0.31 (0.22)	0.63 (0.53)
V3.A	1.00 (0.95)	0.71 (0.66)	1.41 (1.36)	0.99 (0.77)	0.99 (0.86)
V3.B	0.77 (0.77)	0.54 (0.54)	1.08 (1.10)	0.13 (0.15)	0.44 (0.48)
V3.C	0.85 (0.83)	0.59 (0.57)	1.24 (1.21)	0.40 (0.33)	0.65 (0.58)
V3.D	0.78 (0.73)	0.54 (0.50)	1.13 (1.08)	0.19 (0.11)	0.53 (0.41)
V3.M	0.88 (0.85)	0.62 (0.59)	1.26 (1.22)	0.498 (0.38)	0.69 (0.61)
C5b.1	1.16 (1.15)	0.83 (0.82)	1.63 (1.61)	0.38 (0.42)	0.65 (0.61)
C5b.2	1.24 (1.20)	0.89 (0.85)	1.74 (1.71)	0.20 (0.30)	0.53 (0.58)
C5b.A	1.40 (1.39)	1.00 (0.99)	1.96 (1.96)	0.052 (0.058)	0.24 (0.24)
C5b.C	1.12 (1.12)	0.80 (0.80)	1.57 (1.57)	0.50 (0.52)	0.69 (0.68)
C5b.D	1.15 (1.16)	0.81 (0.82)	1.62 (1.65)	0.43 (0.41)	0.65 (0.61)
C5b.MB	1.24 (1.21)	0.88 (0.85)	1.74 (1.73)	0.23 (0.28)	0.53 (0.58)
CD4	1.10 (1.03)	0.81 (0.76)	1.49 (1.40)	0.54 (0.86)	0.70 (0.86)
ADCC	0.96 (0.92)	0.68 (0.65)	1.35 (1.30)	0.80 (0.64)	0.83 (0.81)
Avidity	0.94 (0.76)	0.65 (0.50)	1.34 (1.15)	0.72 (0.19)	0.77 (0.51)
IgA	**1.39**	**1.00**	**1.94**	**0.049**	**0.24**
NAb.aggregate	1.08 (0.95)	0.76 (0.66)	1.53 (1.38)	0.65 (64)	0.75 (0.86)
gp70V1V2.B (case A)	**0.71 (0.63)**	**0.49 (0.43)**	**1.02 (0.92)**	**0.063 (0.02)**	**0.24 (0.13)**

^1^ Designations indicate specific regions of gp120 (C1, V2, V3, C5) followed by genetic subtype or CRF of the consensus peptide (subtypes A, B, C, D; 1= CRF01-AE, 2= CRF02_AG, M= group M, aggregate= all subtypes and CRFs combined). In some cases the peptide sequence was identical for multiple subtypes and CRFs (e.g., C1.MABCD2). CD4, ADCC, Avidity, IgA, NAb.aggregate and gp70V1V2.B (case A) are primary variables from the original RV144 correlates study [32].

^2^ Odds ratio per one standard deviation for each aggregate peptide-specific IgG variable and other primary immune variables. Numbers in parentheses correspond to estimates from the model adjusting for IgA level.

^3^ Lower and upper confidence intervals. Numbers in parentheses correspond to estimates from the model adjusting for IgA level.

^4^ Variables with p-values <0.05 are shown in boldface type for the entire row. Numbers in parentheses correspond to estimates from the model adjusting for IgA level.

^5^ Multiple correction (q-value) is done across all variables. Numbers in parentheses correspond to estimates from the model adjusting for IgA level.

### Associations between peptide-specific IgG and primary immune variables in RV144

We sought to determine whether any peptide-specific IgG variables showed a significant interaction with the primary immune variables in RV144. As shown in Table 3, the V3.1 (CRF01_AE) IgG variable interacted significantly with several primary immune variables, including ADCC (p=0.01), avidity (p=0.002), IgA (p=0.002) and aggregate neutralizing Ab (p=0.0003). In addition, the V3.A, V3.C, V3.D and V3.M IgG variables all showed a significant interaction with aggregate neutralizing Abs (p=0.04, p=0.03, p=0.01, p=0.04, respectively). The V3.2, V3.A and V3.M IgG variables showed a significant interaction with the CD4 variable (p=0.05, p=0.04, p=0.05, respectively), whereas the V3.C and V3.D IgG variables interacted significantly with the IgA variable (p=0.01, p=0.03, respectively). The only other significant interactions were seen with the C1.1 and C1.MABCD2 IgG variables, both of which interacted with the CD4 variable (p=0.05 and p=0.03, respectively). After FDR adjustment, the V3.1 (CRF01_AE) IgG interaction remained significant for avidity (q=0.07), IgA (q=0.07) and aggregate neutralizing Ab (q=0.04) (Table 4). Also, the V3.D IgG interaction remained significant for aggregate neutralizing Ab (q=0.17), whereas a trend was seen for the V3.C IgG interaction with IgA (q=0.21).

**Table 3:** P-values for interactions between RV144 peptide-specific IgG and primary immune variables, without FDR adjustment.

	**p-values^1^**					
**IgG variable^2^**	**CD4**	**ADCC**	**Avidity**	**IgA**	**NAb Aggr.**	**gp70V1V2**
C1a.1	**0.05**	0.83	0.27	0.24	0.31	0.32
C1a.MABCD2	**0.03**	0.11	0.64	0.59	0.63	0.91
V2.1	0.19	0.18	0.26	0.91	0.31	0.97
V2.A	0.06	0.29	0.41	0.69	0.49	0.50
V2.B	0.62	0.79	0.37	0.70	0.39	0.79
V2.D	0.28	0.57	0.95	0.66	0.82	0.70
V2.MC2	0.18	0.09	0.34	0.90	0.39	0.73
V3.1	0.07	**0.01**	**0.002**	**0.002**	**0.0003**	0.07
V3.2	**0.05**	0.16	0.32	0.87	0.46	0.42
V3.A	**0.04**	0.16	0.08	0.16	**0.04**	0.24
V3.B	0.69	0.14	0.25	0.80	0.16	0.38
V3.C	0.07	0.11	0.35	**0.01**	**0.03**	0.23
V3.D	0.21	0.10	0.08	**0.03**	**0.01**	0.30
V3.M	**0.05**	0.15	0.28	0.27	**0.04**	0.66
C5b.1	0.68	0.93	0.53	0.59	0.31	0.20
C5b.2	0.80	0.53	0.17	0.31	0.23	0.38
C5b.A	0.82	0.59	0.79	0.75	0.32	0.50
C5b.C	0.96	0.32	0.23	0.41	0.16	0.89
C5b.D	0.44	0.55	0.57	0.43	0.23	0.86
C5b.MB	0.70	0.52	0.45	0.37	0.13	0.81

^1^ Interactions with p-values ≤0.05 are in boldface type. Those with q values <0.2 are underlined. CD4, ADCC, Avidity, IgA, NAb.aggregate and gp70V1V2.B (case A) are primary variables from the original RV144 correlates study [32].

^2^ Designations indicate specific regions of gp120 (C1, V2, V3, C5) followed by genetic subtype of the consensus peptide (1= CRF01-AE; 2= CRF02_AG, M=group M).

**Table 4:** Q-values for interactions between RV144 peptide-specific IgG and primary immune variables.

	**q-values^1^**					
**IgG variable^2^**	**CD4**	**ADCC**	**Avidity**	**IgA**	**Nab Aggr.**	**gp70V1V2**
C1a.1	0.40	0.91	0.64	0.64	0.64	0.64
C1a.MABCD2	0.39	0.55	0.84	0.81	0.84	0.94
V2.1	0.62	0.60	0.64	0.94	0.64	0.97
V2.A	0.43	0.64	0.69	0.85	0.76	0.77
V2.B	0.83	0.91	0.68	0.85	0.68	0.91
V2.D	0.64	0.81	0.97	0.85	0.91	0.85
V2.MC2	0.61	0.52	0.66	0.94	0.68	0.88
V3.1	0.47	0.26	**0.07**	**0.07**	**0.04**	0.47
V3.2	0.40	0.59	0.64	0.93	0.72	0.69
V3.A	0.40	0.59	0.47	0.59	0.40	0.64
V3.B	0.85	0.59	0.64	0.91	0.59	0.68
V3.C	0.47	0.55	0.66	0.21	0.39	0.64
V3.D	0.64	0.52	0.47	0.39	**0.17**	0.64
V3.M	0.40	0.59	0.64	0.64	0.40	0.85
C5b.1	0.85	0.95	0.77	0.81	0.64	0.62
C5b.2	0.91	0.77	0.59	0.64	0.64	0.68
C5b.A	0.91	0.81	0.91	0.89	0.64	0.77
C5b.C	0.97	0.64	0.64	0.69	0.59	0.94
C5b.D	0.72	0.80	0.81	0.71	0.64	0.93
C5b.MB	0.85	0.77	0.72	0.68	0.59	0.91

^1^ Interactions with q-values <0.2 are in boldface type. CD4, ADCC, Avidity, IgA, NAb.aggregate and gp70V1V2.B (case A) are primary variables from the original RV144 correlates study [32].

^2^ Designations indicate specific regions of gp120 (C1, V2, V3, C5) followed by genetic subtype of the consensus peptide (1= CRF01-AE; 2= CRF02_AG, M=group M).

### V3 peptide-specific IgG as an additional correlate of reduced infection risk in RV144

**Figure 7:**
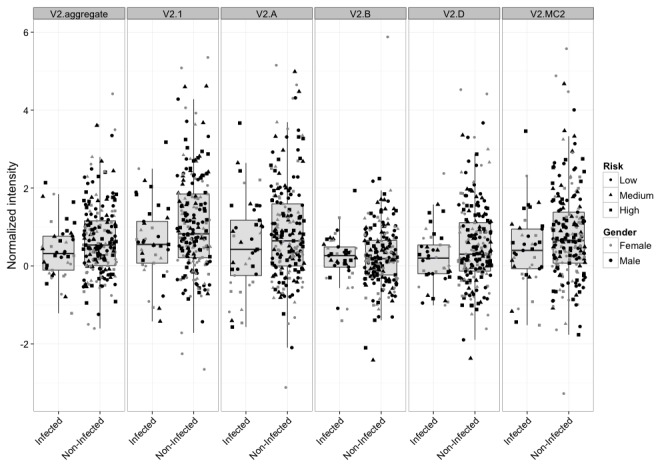
Complementary cumulative distribution function (ccdf) for V3-CRF01_AE (V3.1) peptide binding in RV144. The ccdf is broken down by infection status and by dichotomized IgA, neutralizing Ab and Avidity levels. Low/High dichotomized levels were defined by dividing the responses into two equal groups around the median. For a given V3.1 value, the ccdf gives the proportion of individuals who have a response above that value. At low levels of IgA, neutralizing Ab and Avidity, the infected groups have lower ccdf for nearly all values, supporting our correlates analysis. This pattern is inversed at high levels of IgA, neutralizing Ab and Avidity, supporting our interaction analysis.

Because the V3 CRF01_AE (V3.1) IgG response in RV144 interacted with multiple RV144 primary immune variables when predicting infection risk (Tables 3 and 4), we estimated its effect and significance when fixing the value of the interacting variables (Table 5, Figure 7). Higher levels of V3 CRF01_AE-specific IgG showed a significant inverse correlation with infection risk in subjects who were in the lower 20% and 50% of IgA values (OR= 0.31, p=0.002, q=0.02; OR=0.49, p= 0.007, q=0.04, respectively). They also showed an inverse correlation with risk in subjects who were in the lower 20% and 50% of aggregate neutralizing Ab values (OR=0.30, p=0.001, q=0.01; OR= 0.50, p=0.008, q=0.04, respectively), and in subjects who were in the lower 20% of ADCC values (OR=0.54, p=0.032, q=0.10) and avidity values (OR= 0.51, p=0.017, q=0.06). No other significant correlations were seen. These analyses are the first to demonstrate a correlation between V3-specific IgG and the risk of acquiring HIV-1-infection in RV144.

**Table 5:** Estimated odds ratio for the V3 CRF01-AE IgG variable at different levels of primary immune variables in RV144.

	**V3 CRF01-AE IgG variable**					
**Primary variable^1^**	**Percentile**	**Odds Ratio^2^**	**CI lower^3^**	**CI upper^3^**	**p-value^4^**	**q-value^4^**
CD4	20%	0.66	0.41	1.05	0.080	0.20
	50%	0.74	0.50	1.11	0.147	0.30
	80%	0.92	0.63	1.36	0.686	0.73
ADCC	20%	**0.54**	0.30	0.95	**0.032**	**0.10**
	50%	0.71	0.46	1.10	0.123	0.28
	80%	1.02	0.68	1.53	0.934	0.93
Avidity	20%	**0.51**	0.30	0.89	**0.017**	**0.06**
	50%	0.83	0.56	1.23	0.358	0.59
	80%	1.16	0.77	1.73	0.484	0.67
IgA	20%	**0.31**	0.15	0.64	**0.002**	**0.02**
	50%	**0.49**	0.29	0.83	**0.007**	**0.04**
	80%	0.92	0.61	1.38	0.687	0.73
gp70V1V2	20%	0.71	0.42	1.18	0.187	0.34
	50%	0.90	0.60	1.33	0.580	0.73
	80%	1.21	0.77	1.91	0.405	0.61
NAb-Aggregate	20%	**0.30**	0.15	0.60	**0.001**	**0.01**
	50%	**0.50**	0.30	0.83	**0.008**	**0.04**
	80%	0.90	0.58	1.39	0.632	0.73

^1^ CD4, ADCC, Avidity, IgA, NAb.aggregate and gp70V1V2.B (case A) are primary variables from the original RV144 correlates study [32].

^2^ Odds ratio per one standard deviation for each IgG aggregate variable and other primary immune variables.

^3^ Lower and upper confidence intervals.

^4^ Variables with p-values <0.05 and q values <0.2 are shown in boldface type.

## Discussion

Overlapping peptide arrays spanning the entire gp160 of all major genetic subtypes of HIV-1 were used here to identify linear B cell epitopes commonly recognized by plasma IgG from individuals who were either HIV-1-infected or gp120-vaccinated. Epitopes in the crown of the V3 loop and the C-terminal region of C5 were dominant in both cases. C1 and V2 of gp120 contained additional dominant epitopes for vaccine-elicited responses, while the gp41 ID and HR-1 regions contained additional dominant epitopes for HIV-1-infected individuals (gp41 was not in the vaccines). All of these epitopes have been described previously as immunodominant in HIV-1-infected individuals [65-70] but only limited work has been done in vaccine recipients [71-74]. These linear epitopes are presumably unrelated to most broadly neutralizing Abs that bind discontinuous conformation-dependent epitopes [7-18]. Some linear epitopes in V2 and V3 are common targets for neutralization of Tier 1 viruses [75,76]; however, such readily-neutralized viruses are thought to be rare in nature [77]. Thus, for the most part, the Abs detected here are likely to be non-neutralizing for a majority of circulating HIV-1 variants. They remain interesting because of their potential to bind epitopes on infectious virions [22], or on the surface of infected cells [23], where their FcR-mediate effector functions might have value for vaccines [24-28]. Indeed, many of the epitopes identified here appear to be exposed on the surface of cell free virions and infected cells [78-81].

Our study included case-control samples from RV144 with the goal of seeking correlates of infection risk in vaccine recipients. The V2 region of gp120 has been implicated as a site of immune pressure and as a target for plasma IgG responses that correlated with a reduced risk of infection in RV144 [32,35]. This IgG correlate was identified by using a gp70-V1V2 fusion protein that did not differentiate responses among V1 and V2. Moreover, the correlate was not seen with linear and cyclized V2 peptides [32], making it possible that the key epitope(s) required a conformation that was only present on the fusion protein. Indeed, recent evidence suggests that the IgG response in RV144 targeted a structurally polymorphic region of V2 that is capable of existing in helical, loop and β strand conformations when bound by different monoclonal Abs [36]. Our results showing that plasma IgG to linear V2 peptides correlates with a lower risk of infection in RV144 indicate that conformation in the setting of a gp70-V1V2 fusion protein is not an absolute requirement to detect the correlate. The results further indicate that V2 was the target of the IgG correlate detected with the gp70-V1V2 antigen, although we do not exclude the possibility of two V2 correlates, one that is linear and another that is conformation-dependent. Failure to detect the V2 IgG correlate with other linear and cyclic V2 peptides used in the initial RV144 correlates study [32] might be explained by differences in peptide sequence, size or structure, or by differences in assay methodologies.

The lack of detection of a correlate with subtype B V2 linear peptides is in contrast to the correlation seen with a subtype B gp70-V1V2 protein [32]. In this case of clade mismatch between the detection antigen and the major subtype of circulating virus in the vaccine cohort, a key structure in the context of gp70-V1V2 might be required. Our results with linear V2 peptides do however agree with a recent follow-up study that found a significant correlation for IgG to V1V2 of subtypes C and AE, and a trend toward a correlation for IgG to V1V2 of subtype A [82].

Notably, the V2-specific IgG response in the non-protective Vax003 trial was at least as strong as in RV144, including the V2-CRF01_AE response. Thus, robust V2 Ab responses are not necessarily an indication that a vaccine will be protective. For example, V2-specific Abs might be more effective against heterosexual transmission, as in RV144, than they are in higher-risk injection drug users who participated in Vax003. Indeed, a higher frequency of multiple variant transmission was seen in Vax003 placebo recipients than in historical heterosexual cohorts (K. Bar et al., CROI 2012, #F128), suggesting that higher risk groups present a greater challenge for vaccine protection. Another important consideration is the major IgG subclass of the V2 response. Env-specific and V1V2-specific IgG3 response rates were shown to be somewhat lower in Vax003 than in RV144, where the V1V2-specific IgG3 response in RV144 correlated with a decreased risk of HIV-1 infection [83]. Finally, it is noteworthy that the V2 response in Vax004 and HIV-1-infected subjects was relatively weak, including subjects who were infected with CRF01_AE viruses. Thus, gp120 immunogens can induce V2-specific IgG responses that are infrequent in HIV-1-infected individuals, are dependent on the type of immunogen used and, under certain conditions, are associated with a lower risk of HIV-1 infection in vaccine recipients.

In addition to V2, serum IgG to V3 CRF01_AE was another correlate of lower infection risk in RV144; however, this latter correlation was only seen in vaccine recipients who had lower levels of other Abs, including Env-specific IgA, neutralizing Abs and to a lesser extent, Ab avidity and ADCC activity. Higher levels of Env-specific IgA directly correlated with infection rate in RV144 [32], suggesting that IgA mitigated the protective effects of other Abs [84]. A similar phenomenon has been observed in several human disorders [85-88].

The interactive V3 IgG correlate appears more complex because neutralizing Abs, avidity and ADCC alone were not direct correlates of infection rate [32]. Notably, a substantial fraction of RV144 vaccine-elicited neutralizing Abs to Tier 1 viruses as measured in TZM-bl cells mapped to linear epitopes in the crown of V3 [31]. This agrees with the direct interaction between V3 IgG and neutralizing Ab response to Tier 1 viruses (Table 3), and suggests that any underlying protective mechanism associated with the V3 IgG correlate does not involve neutralization of Tier 1 viruses as measured in the TZM-bl assay. The interactive V3 IgG correlate might be explained by multiple Abs of different isotypes and effector functions competing for overlapping epitopes. Notably, the Ab response after final inoculation in RV144 was relatively weak compared to the response after four inoculations with AIDSVAX B/E in Vax003 [31], indicating that the Ab response in RV144 had not matured to its full potential. This generates the hypothesis that a particular balance of Abs was present during an intermediate stage of maturation of the vaccine-elicited Ab response in RV144 that favored protection. Additional studies are needed to dissect the different V3-specific IgG epitopes, subclasses, glycans and functions in RV144. Two V3-specific monoclonal Abs from RV144 have been described (CH22, CH23); both exhibit potent neutralizing activity against one or more Tier 1 viruses and bind V3 peptides that overlap the V3 reactive region identified here [31]. In addition, a multitude of monoclonal Abs to the crown of the V3 loop have been derived from HIV-1-infected individuals that neutralize most Tier 1 and some Tier 2 viruses from many subtypes [14,89,90]. Notably, these are specific for the crown of the V3 loop and are not glycan-dependent as are the bnAbs that target the 332 glycan-dependent epitope at the base of the V3 loop. Though strong V3 peptide-binding IgG responses were seen in the absence of protection in Vax003 and Vax004, both trials were conducted in higher risk groups, and Env-specific IgA levels have not been evaluated in either trial.

Our assessment of IgG responses by using overlapping peptides spanning the entire gp160 of all major subtypes and CRFs of HIV-1 is the most extensive of its kind to date. The results illustrate that a number of linear epitopes are targets for Abs that do not neutralize Tier 2 viruses, where it is possible that these antibodies possess FcR-mediated antiviral effector functions. Structural considerations reveal that most of the reactive peptide regions identified in this study occur in flexible regions that can easily adopt many different conformations depending on whether they are part of gp120 protein or fragmented, and their conformations can be induced by antibodies. Accordingly, many of them are found outside the gp120 core. Even those that appear within the gp120 core tend to occur in the inner-domain or near the C-terminal end of gp120, regions that are prone to significant conformational changes. It is known that the outer domain core is relatively stable and has a well-defined structure. Only a single peptide region (C5a) was found in this region. There are several possible reasons why these flexible regions were targets for antibodies. First, they have the ability to take on multiple conformations, including those that create the antigenic structure. Second, the potential to adopt a variety of conformations is much higher for peptides in regions such as V2 and V3 because they exhibit both structural and sequence variability. As in the case of V2 peptides, conformational plasticity coupled with sequence variation enables these peptides to accommodate multiple binding modes with antibodies. Thirdly, since their conformational variability is not constrained as a fragmented peptide or as part of gp120 protein, their immunologic properties may not be compromised greatly when fragmented out of the gp120 protein context.

Although the importance of these epitopes is uncertain at this time, the correlations we identified in RV144 generate the hypothesis that protection in RV144 involved a complex balance of multiple immune responses, including a requirement for IgG responses against linear epitopes in the V2 and/or V3 loops of gp120. These IgG responses might be protective on their own, or they may be surrogates of other protective antibodies, such as those in the mucosal compartment [91-93]. Should the IgG peptide binding responses identified here prove important in the future, we note that gp41 contains additional linear epitopes that might be worthy of consideration for vaccine design. Alternatively, removal of these dominant linear gp120 and gp41 epitopes from candidate vaccine immunogens might help focus the Ab response on conformational epitopes that elicit broadly neutralizing antibodies.

## Supporting Information

Table S1 **Sequence and annotation of overlapping gp160 peptides.** ^1^Designations are: sequence number_subtype(s) and/or CRF(s) _number of subtypes and CRFs containing the sequence. Thus, 278_MBD12_5 is peptide region number 278 with a sequence that is shared with group M, subtype B, subtype D, CRF01 and CRF02, for a total of 5 subtypes/CRFs.
(XLSX)
